# Diacylglycerol kinase ζ inhibits myocardial atrophy and restores cardiac dysfunction in streptozotocin-induced diabetes mellitus

**DOI:** 10.1186/1475-2840-7-2

**Published:** 2008-02-04

**Authors:** Olga Bilim, Yasuchika Takeishi, Tatsuro Kitahara, Takanori Arimoto, Takeshi Niizeki, Toshiki Sasaki, Kaoru Goto, Isao Kubota

**Affiliations:** 1 Department of Cardiology, Pulmonology and Nephrology, Yamagata University School of Medicine, Yamagata, Japan; 2 Department of Anatomy and Cell Biology, Yamagata University School of Medicine, Yamagata, Japan

## Abstract

**Background:**

Activation of the diacylglycerol (DAG)-protein kinase C (PKC) pathway has been implicated in the pathogenesis of a number of diabetic complications. Diacylglycerol kinase (DGK) converts DAG to phosphatidic acid and acts as an endogenous regulator of PKC activity. Akt/PKB is associated with a downstream insulin signaling, and PKCβ attenuates insulin-stimulated Akt phosphorylation.

**Methods and Results:**

We examined transgenic mice with cardiac-specific overexpression of DGKζ (DGKζ-TG) compared to wild type (WT) mice in streptozotocin-induced (STZ, 150 mg/kg) diabetic and nondiabetic conditions. After 8 weeks, decreases in heart weight and heart weight/body weight ratio in diabetic WT mice were inhibited in DGKζ-TG mice. Echocardiography at 8 weeks after STZ-injection demonstrated that decreases in left ventricular end-diastolic diameter and fractional shortening observed in WT mice were attenuated in DGKζ-TG mice. Thinning of the interventricular septum and the posterior wall in diabetic WT hearts were blocked in DGKζ-TG mice. Reduction of transverse diameter of cardiomyocytes isolated from the left ventricle in diabetic WT mice was attenuated in DGKζ-TG mice. Cardiac fibrosis was much less in diabetic DGKζ-TG than in diabetic WT mice. Western blots showed translocation of PKCβ and δ isoforms to membrane fraction and decreased Akt/PKB phosphorylation in diabetic WT mouse hearts. However in diabetic DGKζ-TG mice, neither translocation of PKC nor changes Akt/PKB phosphorylation was observed.

**Conclusion:**

DGKζ modulates intracellular signaling and improves the course of diabetic cardiomyopathy. These data may suggest that DGKζ is a new therapeutic target to prevent or reverse diabetic cardiomyopathy.

## Introduction

Diabetes mellitus is a serious medical problem. It is complicated by progressive cardiovascular diseases, including arteriosclerosis, myocardial infarction, and congestive heart failure. Indeed, cardiovascular complications are now the leading cause of morbidity and mortality in diabetic patients. The Framingham study has demonstrated the increased incidence of congestive heart failure in diabetes mellitus (2.4 fold in male and 5 fold in female) independent of age, hypertension, obesity, cardiovascular diseases and hyperlipidemia [1].

Experimental and clinical studies have suggested that diabetic state causes a specific diabetic cardiomyopathy independent of vascular complications. This cardiomyopathy is characterized by structural, metabolic and functional damage to the heart and may be responsible for the high incidence of cardiac dysfunction and mortality in both types 1 and 2 diabetes mellitus. Multiple mechanisms have been implicated in glucose-mediated damage of cardiomyocytes. Of these, activation of protein kinase C (PKC) through the *de novo *synthesis of diacylglycerol (DAG) has been increasingly recognized as an early and common mechanism leading to cardiac dysfunction and remodeling in diabetes [2-4]. Diacylglycerol kinase (DGK) is suggested to attenuate DAG-induced PKC activation through the phosphorylation of this second messenger and conversion to phosphatidic acid (PA) [5,6]. A previous study has indicated that three DGK isoforms (DGKα, ε, and ζ) are expressed in the rat myocardium, and the DGKζ isoform is predominant in rodent [7].

We have previously demonstrated that overexpression of DGKζ, in both cultured rat neonatal cardiomyocytes and in vivo mouse hearts, prevents pathological activation of PKC and improves the course of left ventricular remodeling in infarcted myocardium [8], angiotensin II and phenylephrine-induced cardiac hypertrophy [9], and pressure overloaded heart [10]. However, the effects of DGKζ on diabetes-induced cardiac structural changes and cardiomyocyte signal transduction have not been previously examined. In the present study, we tested the hypothesis that DGKζ attenuates changes in cardiac structure and function in response to hyperglycemia. We employed the mouse model of diabetes mellitus by intraperitoneal injection of streptozotocin (STZ) and examined whether DGKζ inhibits hyperglycemia-induced activation of signaling pathways and cardiac dysfunction in STZ-induced diabetes mellitus.

## Methods

### Animals and experimental protocols

DGKζ-TG mice were created in our institution as previously reported [9], and DGKζ-TG mice and wild type littermates (WT) were used in the present study. Mice were housed under specific-pathogen-free conditions in a facility with a 12 hr-12 hr light-dark cycle and were given free access to water and standard rodent chow. All experimental procedures were performed according to the animal welfare regulations of Yamagata University School of Medicine, and the study protocol was approved by the Animal Subjects Committee of Yamagata University School of Medicine. The investigation conformed to the *Guide for the Care and Use of Laboratory Animals*, published by the National Institutes of Health.

Diabetes was induced in 8–10 weeks old weighing 20–25 g male mice by a single intraperitoneal injection of STZ (Sigma Aldrich, Tokyo, Japan), dissolved in 10 mM citrate buffer (pH 4.5). Control mice were treated with the same volume of citrate buffer. Mice were fasted 5 hours prior to injection. Diabetes was confirmed at 2 weeks after STZ injection by measuring the glucose concentration of peripheral blood obtained from the tail vein using NIPRO FreeStyle blood glucose monitoring system (Nipro Corporation, Osaka, Japan). Afterwards the blood glucose levels and body weight of mice were monitored weekly. The mice having plasma glucose levels more than 300 mg/dl were classified as diabetic and used for the present study.

### Echocardiography

Transthoracic echocardiography was recorded as described previously [11-13] with an FFsonic 8900 (Fukuda Denshi Co., Tokyo, Japan) equipped with a 13-MHz phased-array transducer at baseline and 8 weeks after STZ injection. Left ventricular internal dimensions at end-systole and end-diastole (LVESD and LVEDD), posterior wall thickness (PW) and interventricular septal wall thickness (IVS) were measured digitally on the M-mode tracings and averaged from at least 3 cardiac cycles [11-13]. Left ventricular fractional shortening (LVFS) was calculated as [(LVEDD -LVESD)/LVEDD] × 100 (%).

### Morphological examinations

After echocardiography, mice were sacrificed, coronary arteries were retrogradely perfused with saline, and the heart and lungs were excised and weighed. The heart was fixed with a 10% solution of formalin in phosphate buffered saline and embedded in paraffin [11,12]. Three micron sections were evaluated using standard protocols for hematoxylin eosin staining to determine left ventricular cross-sectional areas and Masson trichrome staining for fibrosis as previously described [12]. Transverse sections were captured digitally, and cardiomyocyte cross-sectional area was measured using NIH ImageJ 1.37 V (Bethesda, MD, USA). Mean cardiomyocyte cross-sectional areas were calculated by averaging the measurements of 100 cells from sections [11,12]. To assess the degree of fibrosis, the sections stained with Masson trichrome were scanned with computer-assisted videodensitometry, and the images from at least 10 fields for each heart were analyzed as described previously [11,12]. The fibrosis fraction was obtained by calculating the ratio of Masson trichrome stained connective tissue area (stained blue) to total myocardial area (stained red) with an image analysis software.

### Western blotting

The left ventricular tissue for Western blotting was immediately frozen in liquid nitrogen and stored at -80°C until use. Total protein was extracted with ice-cold lysis buffer as discribed previously [14-16]. To examine phosphorylation activity of Akt/PKB, Western blotting was performed with an anti-phosphospecific Akt (Ser473) antibody, which detects Akt only when phosphorylated at Ser473 (Cell Signalling, Denvers, MA, USA) as reported previously [17]. The same membranes were then reprobed with nonspecific anti-Akt antibody to quantify the amount of Akt protein.

Translocation of PKC isoforms was examined by quantitative immunoblotting using isoform-specific antibodies (mouse monoclonal anti-PKC-α, -β, -δ, and -ε, Santa Cruz Biotechnology, Santa Cruz, CA) in membrane and cytosolic fractions prepared from the left ventricular myocardium as described previously [14-16]. Immunoreactive bands were detected by an ECL kit (Amersham Biosciences, Piscataway, NJ), and membrane-to-cytosol ratios of immunoreactivity were used as indices for the extent of translocation of PKC isoforms [14-16].

### Statistical analysis

Continuous variables are reported as mean ± SEM. Effects of STZ injection on morphological parameters, echocardiographic measurements, histological data between WT and TG mice were analyzed by two-way ANOVA followed by a Bonferroni test. A value of P < 0.05 indicated statistical significance.

## Results

### General features and gravimetric data of animals

The general features of diabetic mice and age-matched nondiabetic control mice are summarized in Table 1. Blood glucose concentrations and body weight at baseline were similar among 4 groups (data not shown). Gravimetric parameters as well as blood glucose concentration were not significantly different between control WT and control DGKζ-TG mice at 8 weeks after injection of citrate buffer (Table 1). Intraperitoneal injection of STZ induced diabetes mellitus in both WT and DGKζ-TG mice. At 8 weeks after STZ injection, both diabetic WT and diabetic DGKζ-TG groups had markedly elevated plasma glucose levels compared with control mice (P < 0.01, Table 1). Plasma glucose levels did not differ between diabetic DGKζ-TG and diabetic WT mice. Both diabetic mice had less body weight than control mice (P < 0.01). Diabetic WT and diabetic DGKζ-TG mice at 8 weeks after STZ injection had a remarkably lower absolute heart weight and left ventricular weight than control animals (P < 0.01). However, decreases in absolute heart weight, heart weight/body weight ratio and left ventricular weight/body weight ratio were attenuated in diabetic DGKζ-TG mice compared to diabetic WT mice (P < 0.05).

**Table 1:** General characteristics of DGKζ-TG and WT mice at 8 weeks after STZ injection.

	WT control	DGKζ-TG control	WT STZ	DGKζ-TG STZ
BW, g	32.08 ± 0.70	32.3 ± 0.65	22.37 ± 0.91*	22.8 ± 0.54§
BG, mg/dl	155.4 ± 0.47	157.7 ± 0.54	465.8 ± 0.61*	455.3 ± 0.53§
HW, mg	147 ± 0.02	142 ± 0.01	92 ± 0.02*	110 ± 0.02§ddag
LVW, mg	107 ± 0.01	105 ± 0.01	69 ± 0.01*	77 ± 0.01§
HW/BW ratio, mg/g	4.58 ± 0.13	4.41 ± 0.08	4.00 ± 0.14*	4.44 ± 0.12‡
LVW/BW ratio, mg/g	3.32 ± 0.07	3.24 ± 0.05	3.0 ± 0.09*	3.25 ± 0.08‡

Data are mean ± SEM from 15 mice for each group. BW, body weight; BG, blood glucose; HW, heart weight; LVW, left ventricular weight. *P < 0.01 vs. WT control, ‡P < 0.05 vs. WT STZ, §P < 0.01 vs. DGKζ-TG control.

### Echocardiographic measurements

**Figure 1:**
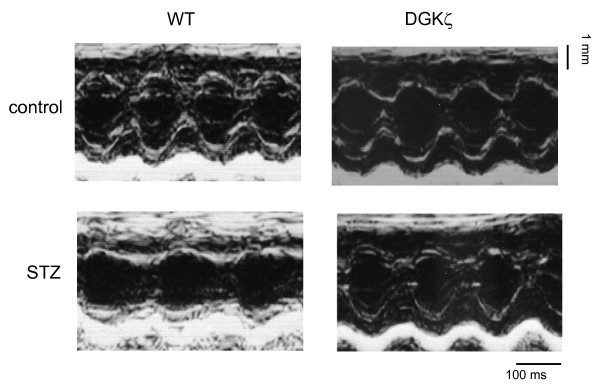
Representative M-mode echocardiograms of WT and DGKζ-TG mice at 8 weeks after injection of STZ or citrate buffer solution.

**Figure 2:**
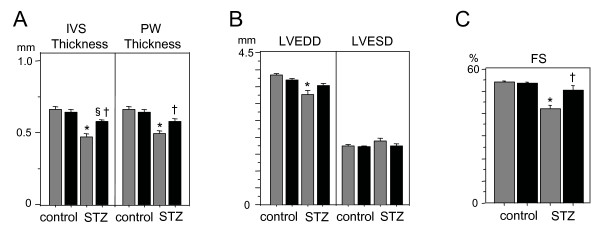
Group data for echocardiographic measurements in WT and DGKζ-TG mice including (A) IVS (interventricular septum) thickness, LVPW (left ventricular posterior wall) thickness, (B) LVEDD (left ventricular end-diastolic dimension), LVESD (left ventricular end-systolic dimension), and (C) FS (fractional shortening). *P < 0.01 vs. WT control, †P < 0.01 vs. WT STZ, §P < 0.01 vs. DGKζ-TG control. Grey bars, WT mice, Black bars, DGKζ-TG mice. (n = 15 for each group).

Echocardiography was performed at baseline and at 8 weeks after STZ injection. Baseline echocardiography demonstrated that heart rate, cardiac dimensions, wall thickness, and fractional shortening were similar between WT and DGKζ-TG mice (data not shown). Representative M-mode echocardiograms following 8 weeks of observation are shown in Figure 1. Thinning of PW and IVS wall thickness in diabetic WT mice were blocked in DGKζ-TG mice (Figure 2). Decreases in LVEDD (P < 0.01 vs. control WT) in diabetic WT mice were not observed in diabetic DGKζ-TG mice (Figure 2). Statistically significant impairment in left ventricular fractional shortening (P < 0.01) was observed in diabetic WT mice compared with control WT mice (Figure 2). However, these functional deteriorations were attenuated in diabetic DGKζ-TG mice (P < 0.01).

### Histological findings

**Figure 3:**
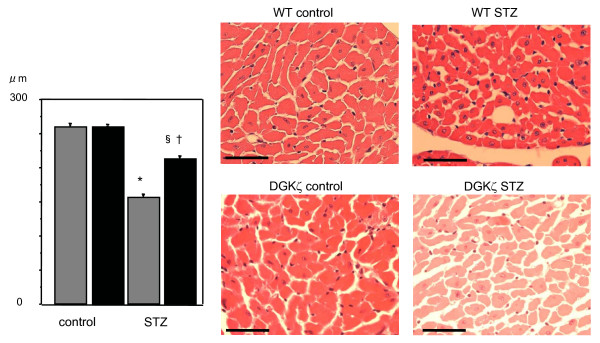
Histological analyses in WT and DGKζ-TG mice at 8 weeks after injection of STZ or citrate buffer solution. Hematoxylin-eosin micrographs showing transverse sections of left ventricular myocardium (× 400, bar = 50 μm). Quantitative analysis of cardiomyocyte cross-sectional area (left bar graph) in WT (grey bars) and DGKζ-TG (black bars) mice. Data were calculated by averaging the measurements at least of 100 cardiomyocytes from each sections. Data were obtained from 8 mice for each group.

**Figure 4:**
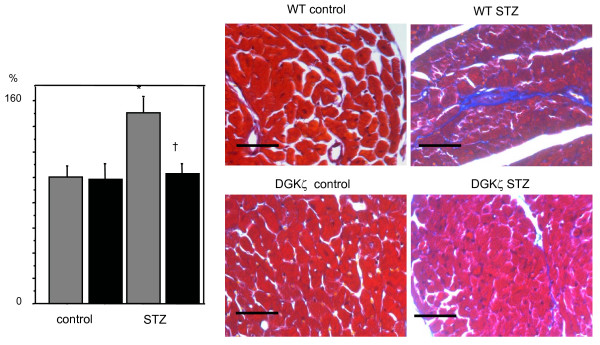
Masson trichrome staining micrographs showing transverse sections of left ventricular myocardium (× 400, bar = 50 μm). Comparisons of the fibrosis fraction (left bar graph) between WT (grey bars) and DGKζ-TG (black bars) mice. The fibrosis fraction was obtained by calculating the ratio of Masson trichrome stained connective tissue area (stained blue) to total myocardial area. Data were calculated by averaging the measurements of 10 fields from each sections. Data were obtained from 8 mice for each group. *P < 0.01 vs. WT control, †P < 0.01 vs. WT STZ, §P < 0.01 vs. DGKζ-TG control.

Figure 2 shows histological observations of the left ventricular myocardium in WT and DGKζ-TG mice following 8 weeks of experiments. A transverse diameter of cardiomyocytes was reduced in both diabetic DGKζ-TG and diabetic WT mice (P < 0.01, Figure 3). However, decreases in cardiomyocyte transverse diameter were attenuated in diabetic DGKζ-TG mice compared to diabetic WT mice (P < 0.01). Interstitial fibrosis was observed in diabetic WT mouse hearts as shown in Figure 4, but the degree of fibrosis was much less in diabetic DGKζ-TG than in diabetic WT mice (P < 0.01).

### Translocation of PKC isoforms

**Figure 5:**
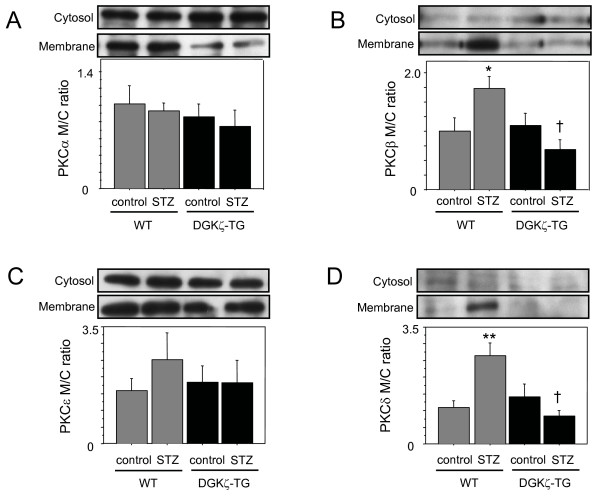
Representative Western blots of PKC-α (A), -β (B), -ε (C), and -δ (D) isoforms in membrane and cytosol fractions and densitometric analysis in WT (grey bars) and DGKζ-TG (black bars) mice at 8 weeks after injection of STZ or citrate buffer solution. Membrane/cytosol ratios of immunoreactivity were used as indices of the extent of PKC isoform translocation. We detected that STZ-induced diabetes caused an increase in the membrane/cytosol ratio of PKC-β and -δ isoforms in WT mouse hearts. However in DGKζ-TG mice, translocation of PKC-β and -δ isoforms was significantly attenuated. Data were obtained from 6 mice for each group. *P < 0.05, **P < 0.01 vs. WT control, †P < 0.01 vs. WT STZ.

As shown in Figure 5, we detected that STZ-induced diabetes caused translocation of PKC β and δ isoforms in WT mouse hearts. However in DGKζ-TG mice, translocation of PKC β and δ isoforms was significantly attenuated (P < 0.01). Statistically significant changes in the membrane/cytosolic ratios of the PKCα and ε isoforms were not observed after STZ in our myocardial preparations.

### Changes in Akt/PKB phosphorylation

**Figure 6:**
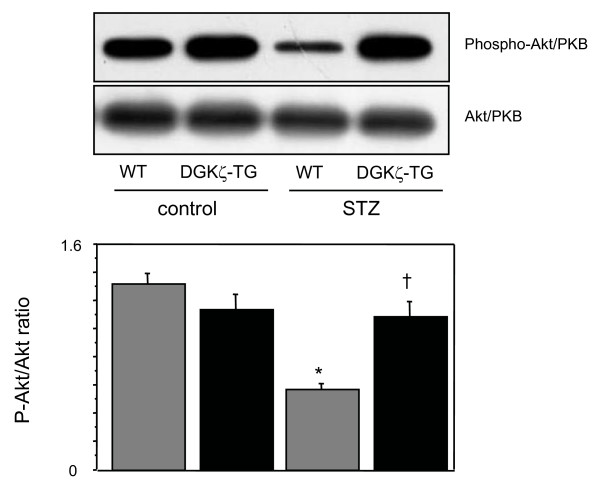
Representative immunoblots of left ventricular extracts with anti- phosphospecific Akt/PKB antibody (upper gel) in WT and DGKζ-TG mice at 8 weeks after injection of STZ or citrate buffer solution. The abundance of Akt protein was demonstrated by immunoblots with an antibody to total Akt (lower gel). Densitometric analyses of Akt phosphorylation were performed using 8 mice for each group. *P < 0.01 vs. WT control, †P < 0.01 vs. WT STZ.

We examined Akt/PKB phosphorylation in diabetic and nondiabetic WT and DGKζ-TG mice. Akt/PKB regulates different cellular processes, including cell growth and glucose metabolism, and is associated with a downstream insulin signaling [18-21]. We supposed that heart atrophy observed in our study may be caused by impairment in Akt/PKB phosphorylation. Changes in phosphorylation activity of Akt/PKB in STZ-induced diabetic hearts in WT and DGKζ-TG mice were examined by Western blotting using anti-phosphospecific Akt/PKB antibody (Figure 6). We documented decreased serine-473 phosphorylation of Akt/PKB in diabetic WT mouse hearts compared to control WT mouse hearts (P < 0.01). However in DGKζ-TG mice, the inhibition of Akt/PKB phosphorylation was not observed after STZ injection (P < 0.01).

## Discussion

### Principal findings

In the present study, using a mouse model STZ-induced diabetic cardiomyopathy, we demonstrated that left ventricular atrophy and left ventricular systolic dysfunction were attenuated in genetically engineered mice with cardiac specific overexpression of DGKζ. Prominent cardiac fibrosis in diabetic WT mice was not observed in DGKζ-TG mice. In this model of hyperglycemia, we found that these changes in diabetic WT mice were associated with activation of PKC in parallel with inhibition of Akt/PKB phosphorylation. However in diabetic DGKζ-TG mice, neither translocation of PKC from cytosol to membrane fraction nor changes in phosphorylation of Akt/PKB was observed.

Previous studies have shown that hyperglycemia increases the PKC content and activity in the hearts and vasculature of experimental diabetic animals and humans through accumulation of *de novo *synthesized DAG [2,22-25]. This DAG-PKC activation plays a key role in the pathogenesis of structural, metabolic and functional damage in the heart and may be responsible for the high incidence of cardiac dysfunction in diabetes mellitus [2,23]. Previously, we have reported that DGKζ may act as an endogenous regulator of the DAG-PKC signaling cascade in mouse cardiomyocytes by controlling cellular DAG levels [9,26]. In the present study, diabetic WT mice demonstrated translocation of PKC β and δ isoforms from cytosol to membrane fraction. This was associated with the decrease of cardiac pump function and increased interstitial fibrosis. However in DGKζ-TG mice, activation of PKC β and δ isoforms (membranous translocation) in response to hyperglycemia was not observed, and prominent deterioration of left ventricular systolic function as well as cardiac fibrosis was not present.

### Diabetic cardiomyopathy

Clinical studies have demonstrated that diabetic cardiomyopathy is manifested with left ventricular hypertrophy associated with systolic/diastolic dysfunction and cardiac fibrosis in diabetic patients [27,28]. In the present study, we observed cardiac atrophy in diabetic WT mice which was determined by decreases of heart weight and heart weight/body weight ratio, decreases of heart size on echocardiography, thinning of left ventricular wall thickness, and decreases of cardiomyocyte transverse diameter. However, most human diabetes belongs to type 2, and a lot of studies with human type 2 diabetes-induced cardiomyopathy are associated with hyperglycemia and hyperinsulinemia [29]. In contrast, STZ-induced diabetes may serve as a model of the type 1 diabetes of human and is associated with severe hyperglycemia in combination with hypoinsulinemia and ketoacidosis [30]. In experimental animal models of STZ-induced diabetic cardiomyopathy, multiple studies demonstrated myocardial atrophy as opposed to hypertrophy with loss of heart weight, reduced cardiomyocyte transverse diameter, loss of contractile proteins and cardiomyocyte dropout [30,31]. Moreover, in recent studies using myocardial biopsy materials from patients with diabetes without hypertension, smaller diameter of cardiomyocytes was observed than in controls without diabetes and hypertension [32]. Cellular mechanisms of diabetes-triggered cardiac atrophy are not clearly understood. Calorie deprivation associated with metabolic disturbance in diabetes and energy production shifted from glucose utilization towards β-oxidation of free fatty acids may cause atrophic alterations in the myocardium [30,33].

### Possible mechanisms

**Figure 7:**
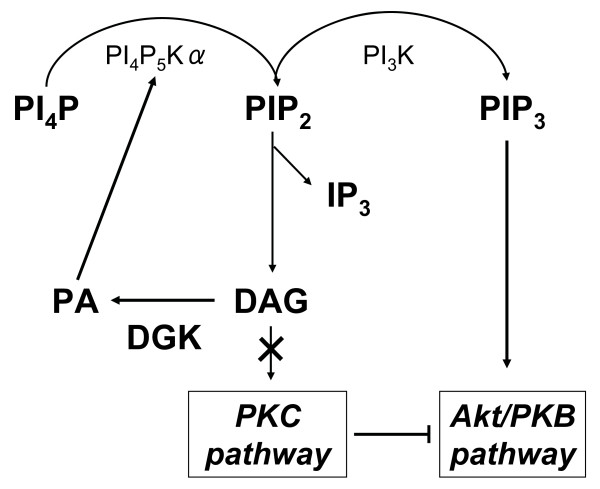
Putative regulating mechanisms of DGK on diabetic cardiomyopathy.

In the present study, diabetic WT mice with myocardial atrophy were associated with decreased phosphorylation of Akt/PKB, the downstream target of insulin action and important kinase for cell growth regulation (Figure 6). In DGKζ-TG mice, hyperglycemia did not suppress Akt/PKB phosphorylation, and cardiac atrophy was not evident compared to diabetic WT mice. We speculate that decreased phosphorylation activity of Akt/PKB, which mediates postnatal heart growth, may account for cardiac atrophy observed in diabetic WT mice [18]. Naruse at al. have shown that PKCβ inhibits insulin-stimulated Akt phosphorylation [34]. PKC negatively regulates Akt activity and reduces both phosphorylation of Akt on Ser-473 and Akt catalytic activity in mouse keratinocyte cell line [35]. Wen et al. have also reported that PKCβ selective inhibitor increases Akt phosphorylation in A549 cells [36]. Thus, PKC might be an inhibitory upstream molecule that regulates Akt phosphorylation. In the present study, hyperglycemia-induced activation of PKCβ was blocked in DGKζ-overexpressing hearts (Figure 5). These data may suggest that PKCβ blockade by DGKζ enhances Akt/PKB phosphorylation in diabetic DGKζ-TG hearts (Figure 7).

By converting cellular DAG to PA, DGK regulates the balance between the two signaling lipids, DAG and PA [37-39]. A previous *in vitro *study has demonstrated that increasing levels of PA modulate phosphatidylinositol 4-phosphate 5-kinase α (PI_4_P_5_Kα) activity [40]. This enzyme catalyzes the synthesis of phosphatidylinositol [4,5]-bisphosphate (PIP_2_) by phosphorylating phosphatidylinositol 4-phosphate (PI_4_P). Phosphoinositide 3-kinase (PI_3_K) converts the plasma membrane lipid PIP_2 _to phosphatidylinositol-3, 4, 5-trisphosphate (PIP_3_), which activates Akt/PKB signaling pathway [18,19,41]. Thus, accumulated PA in DGK overexpressing hearts may activate Akt/PKB through PIP_2 _and PIP_3 _production (Figure 7). Taken together, DGKζ modulates intracellular signaling and improves the course of diabetic cardiomyopathy. In the present study, increased Akt/PKB phosphorylation in DGKζ-TG mice was accompanied by improvement in cardiac function and inhibition of myocardial atrophy. These data are consistent with a previous report that Akt induces enhanced myocardial contractility and cell size in vivo in transgenic mice [42].

### Diacylglycerol kinase (DGK)

One of the best known functional roles of DGK is to regulate PKC activity through DAG metabolism [5,6,37-39]. However, recent reports have suggested that the functional significance of DAG is not restricted to PKC pathway, and DAG also activates several proteins including Ras GRP, protein kinase D, and transient receptor potential proteins. These data suggest that DAG is more widely implicated in cellular events and cellular DAG level is strictly controlled to maintain normal physiological conditions. In addition, phosphatidic acid produced by DGK has signaling functions and serves as a lipid second messenger to regulate a variety of signaling proteins including PKCζ and phospholipase C γ 1. Therefore, DGK is one of the key enzymes closely involved in lipid-mediated cellular signaling events by attenuation of DAG and production of phosphatidic acid. To date, ten DGK isoforms have been identified in mammals such as DGKα, β, γ, δ, ε, ζ, η, θ, ι, and κ, and DGK isoforms are detected in various tissues and cell types, suggesting the importance of this kinase in basic cellular functions [5,6,37-39].

## Conclusion

In conclusion, we demonstrated that DGKζ prevents STZ-induced diabetic cardiomyopathy in an animal model of type 1 diabetes. To our knowledge, this is the first report showing that DGKζ impacts diabetic cardiomyopathy. We also unveil intracellular signaling pathway resulting in formation of diabetic cardiomyopathy. This may provide a novel insight into the prevention and treatment of this pathological process.

## Abbreviations

protein kinase C (PKC); diacylglycerol (DAG); diacylglycerol kinase (DGK); phosphatidic acid (PA); streptozotocin (STZ); transgenic mice with cardiac-specific overexpression of DGKζ (DGKζ-TG); wild type littermates (WT); left ventricular internal dimensions at end-systole (LVESD); left ventricular internal dimension at end-diastole (LVEDD); posterior wall thickness (PW); interventricular septal wall thickness (IVS); left ventricular fractional shortening (LVFS); phosphatidylinositol 4-phosphate 5-kinase α (PI_4_P_5_Kα); phosphatidylinositol [4,5]-bisphosphate (PIP_2_); phosphorylating phosphatidylinositol 4-phosphate (PI_4_P); phosphoinositide 3-kinase (PI_3_K)

## Competing interests

The author(s) declare that they have no competing interests.

## Authors' contributions

All authors have read and approved the final manuscript.

Olga Bilim: conception and design, or acquisition of data, or analysis and interpretation of data, drafting the manuscript. Yasuchika Takeishi: conception and design, interpretation of data, drafting the manuscript and revising it critically for important intellectual content. Tatsuro Kitahara: acquisition of data, or analysis. Taknori Arimoto: acquisition of data, or analysis, Takeshi Niizeki: acquisition of data, or analysis. Toshiki Sasaki: acquisition of data, or analysis. Koru Goto: revising manuscript critically for important intellectual content. Isao Kubota: revising manuscript critically for important intellectual content
